# Supplementary material for: Rotational symmetry breaking in the topological superconductor SrxBi2Se3 probed by upper-critical field experiments
Source: Sci Rep. 2016 Jun 28;6:28632. doi: 10.1038/srep28632 (PMC4923890; doi:10.1038/srep28632)
Supplement: Supplementary Information [file srep28632-s1.pdf]

SUPPLEMENTARY INFORMATION

Rotational symmetry breaking in the topological superconductor $Sr_xBi_2Se_3$ probed by upper-critical field experiments

Y. Pan[1], A. M. Nikitin[1], G. K. Araizi[1], Y. K. Huang[1], Y. Matsushita[2], T. Naka[2] and A. de Visser[1]

[1]*Van der Waals - Zeeman Institute, University of Amsterdam, Science Park 904, 1098 XH Amsterdam, The Netherlands*
[2]*National Institute for Materials Science, Sengen 1-2-1, Tsukuba, Ibaraki 305-0047, Japan*

1. Powder X-ray diffraction

Powder X-ray diffraction patterns have been obtained for $Sr_{0.10}Bi_2Se_3$ and $Sr_{0.15}Bi_2Se_3$. The patterns are in excellent agreement with the calculated pattern for Bi_2Se_3 and the $R\bar{3}m$ space group (see Fig. S1). The lattice parameters (a = 4.137 Å and c = 28.65 Å) are identical for both compositions within the experimental resolution. The tiny extra peaks (black arrows) in the diffraction pattern of $Sr_{0.15}Bi_2Se_3$ points to the presence of a minority impurity phase.

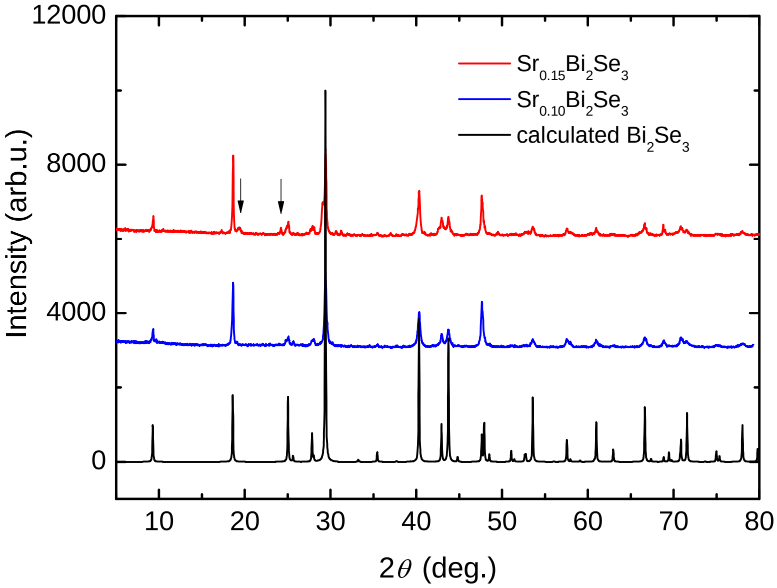

Fig. S1 *Powder X-ray diffraction pattern of $Sr_{0.10}Bi_2Se_3$ (blue line) and $Sr_{0.15}Bi_2Se_3$ (red line) at room temperature. The black line is the calculated pattern for Bi_2Se_3.*

In order to investigate whether $Sr_{0.15}Bi_2Se_3$ undergoes a structural transition the powder X-ray diffraction pattern was measured down to T = 10 K. No change in crystal structure was observed (see Fig. S2). The lattice parameters a and c both show a small decrease with decreasing temperature. The tiny extra peaks (black arrows) in the diffraction pattern point to the presence of a minority impurity phase.

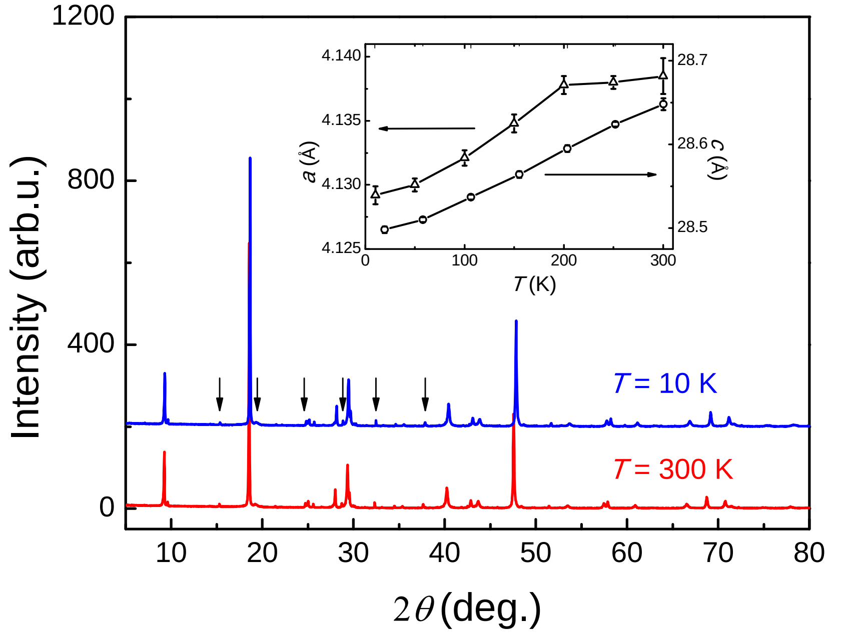

Fig. S2 *Powder X-ray diffraction pattern of* $Sr_{0.15}Bi_2Se_3$ *at 300 K (red line) and at 10 K (blue line). The inset shows the temperature variation of the lattice parameters a and c.*

2. Single-crystal Laue diffraction

Laue diffraction was made on the $Sr_xBi_2Se_3$ crystals to confirm their single-crystalline nature. The Laue diagrams show well defined spots and confirm the trigonal symmetry in the basal plane (see Fig. S3).

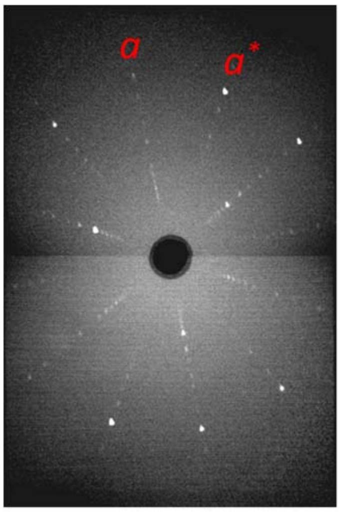

Fig. S3 *Back-scattering Laue diagram of $Sr_{0.15}Bi_2Se_3$ with the incoming beam along the c-axis. The diffraction pattern has trigonal symmetry. The a and a* axis are indicated.*

3. Sample shape and demagnetization factor

The basal-plane transport experiments were performed on thin-bar shaped samples with the current along the long axis. The sizes of the $Sr_{0.10}Bi_2Se_3$ crystal are 1.3×5.5×0.32 mm^3 and of the $Sr_{0.15}Bi_2Se_3$ crystal 1.3×2.3×0.35 mm^3. We have estimated the demagnetization factor N for the field along and perpendicular to the long axis (see Chen *et al.*, IEEE Transactions on Magnetics **38** (2002) 1742). For $Sr_{0.10}Bi_2Se_3$ we estimate $N_{||} = 0.05$ and $N_{\perp} = 0.213$, and for $Sr_{0.15}Bi_2Se_3$ $N_{||} = 0.12$ and $N_{\perp} = 0.20$. For a Type II superconductor a demagnetization factor $N \neq 0$ gives rise to a correction of the internal field $H_{in} = (B_{app}/\mu_0)/(1+ \chi N)$. We conclude these corrections are relatively small for our crystals and we neglect demagnetization effects. Neglecting the effect of N does not have a significant effect on the derived values of the large basal-plane anisotropy of the upper critical field B_{c2}.

4. Sample characterization: resistance and ac-susceptibility

The temperature variation of the resistivity, ρ(*T*), of several $Sr_xBi_2Se_3$ crystals has been measured with a standard 4-probe low-frequency ac-technique in the PPMS. Typical resistivity traces, taken in the temperature range 2-300 K, are shown in Fig. S4. The resistance shows a metallic behavior. The ρ(*T*)-values at low temperature amount to 0.60 and 0.75 mΩcm for $x = 0.10$ and 0.15, respectively, and are in good agreement with the values reported previously (Liu *et al.* JACS **137**, 10512 (2015), Shruti *et al.*, PRB **92**, 020506R (2015)). The superconducting transition temperatures as determined by the midpoints of the transitions are 2.84 K and 2.95 K for $x = 0.10$ and 0.15, respectively. The ac-susceptibility, χ_{AC}, was measured in a driving field of 0.026 mT in the 3-Helium cryostat for several crystals with different sizes. The χ_{AC} data yield superconducting volume fractions of 5-40 % for $x = 0.10$ and 80 % for 0.15, respectively.

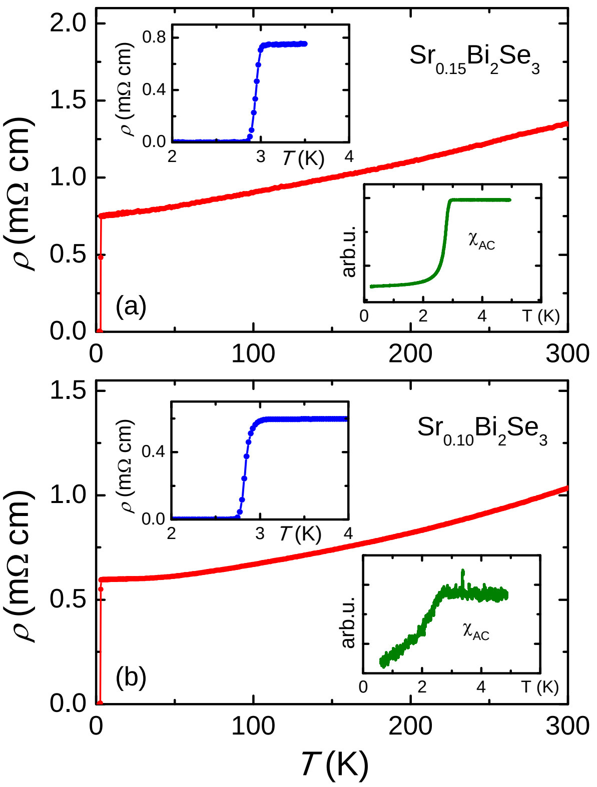

Fig. S4 *Panel (a) and (b): Resistivity of $Sr_xBi_2Se_3$ single crystals with $x = 0.15$ and 0.10, respectively. Upper insets: zoom of the superconducting transition. Lower insets: ac-susceptibility around the superconducting transition.*

5. Superconducting transition in magnetic field of $Sr_{0.10}Bi_2Se_3$

The suppression of the superconducting state by a magnetic field was measured by the resistance as a function of temperature in fixed magnetic fields. The data for $Sr_{0.15}Bi_2Se_3$ taken in the PPMS down to a temperature of 2 K are shown in Fig. S5. The superconducting transition temperatures are determined by the midpoints of the transitions.

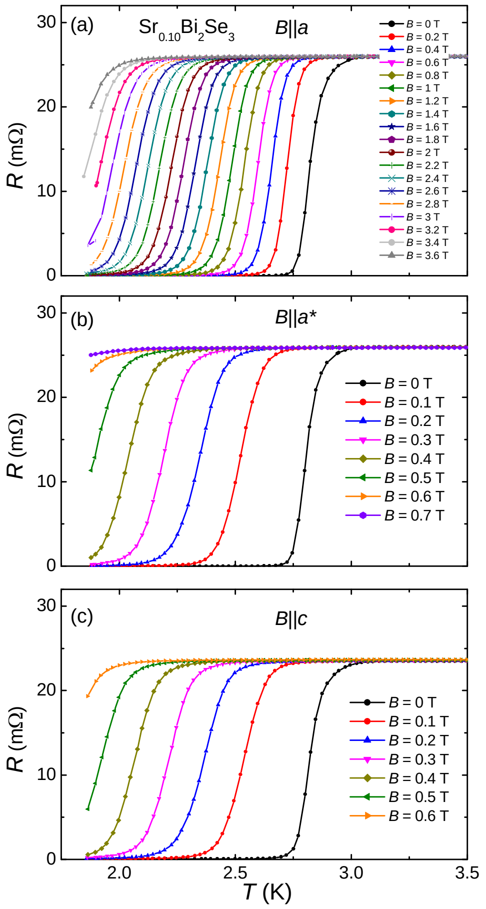

Fig. S5 *Superconducting transition of $Sr_{0.10}Bi_2Se_3$ measured in fixed magnetic fields as indicated. In panel (a), (b) and (c) the B-field is applied along the a, a* and c-axis, respectively.*

6. Superconducting transition in magnetic field of $Sr_{0.15}Bi_2Se_3$

The suppression of the superconducting state by a magnetic field was measured by the resistance as a function of temperature in fixed magnetic fields. The data for $Sr_{0.15}Bi_2Se_3$ taken in the 3-Helium cryostat down to 0.3 K are shown in Fig. S6. The superconducting transition temperatures are determined by the midpoints of the transitions. For $B \parallel a$ the resistance develops a small tail towards $R = 0$, which is attributed to a sample inhomogeneity.

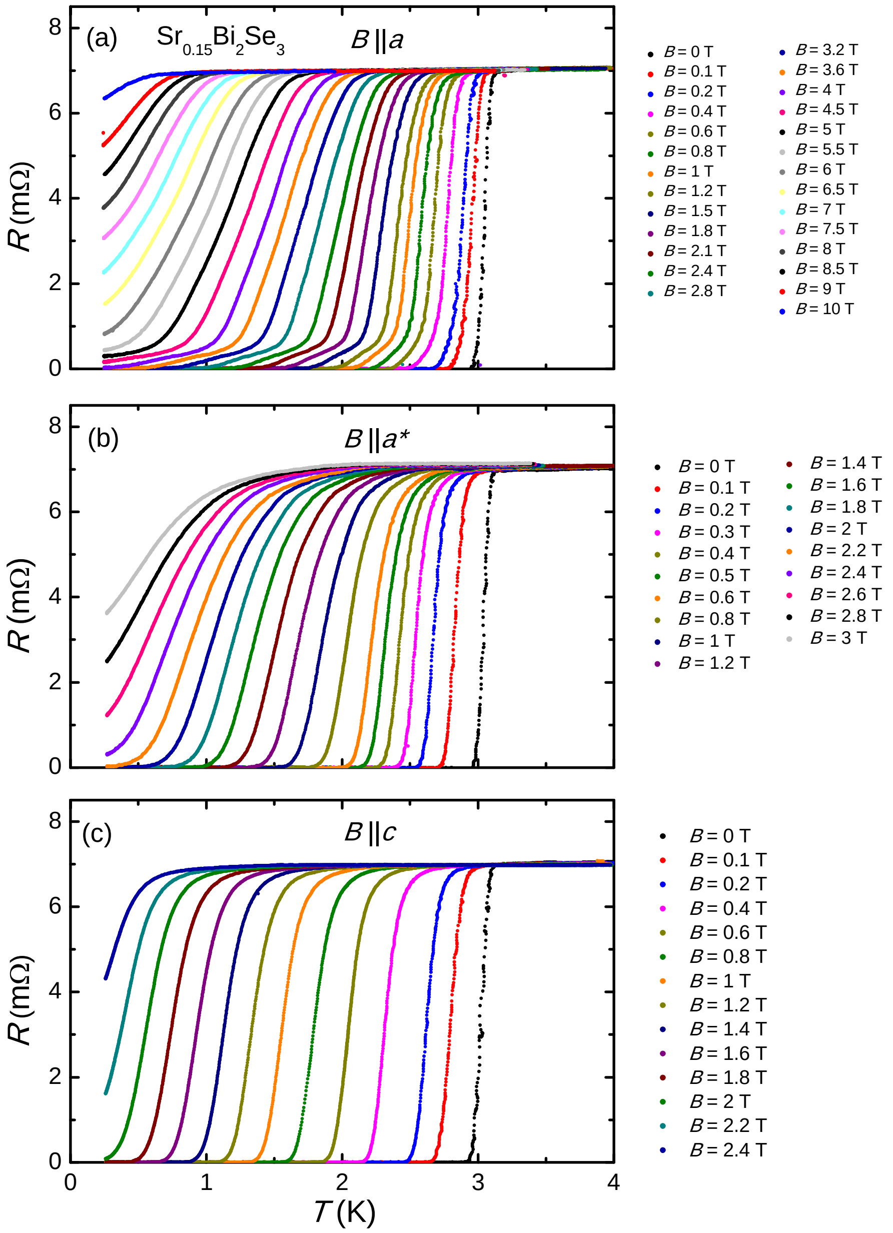

Fig. S6 *Superconducting transition of* $Sr_{0.15}Bi_2Se_3$ *measured in fixed magnetic fields as indicated. In panel (a), (b) and (c) the B-field is applied along the a, a* and c-axis, respectively.*

7. Angular variation of the resistance as a function of magnetic field

The angular variation of $R(B)$ of the $Sr_{0.10}Bi_2Se_3$ crystal was measured in the PPMS at T = 2.0 K, whereas data for $Sr_{0.15}Bi_2Se_3$ were taken at T = 2.0 K in the PPMS and at T = 0.3 K in the 3-Helium cryostat. Selected $R(B)$ curves are shown in Fig. S7. The midpoints of the transitions to the normal state determine $T_c(B)$ and have been used to construct Fig. 3 in the manuscript.

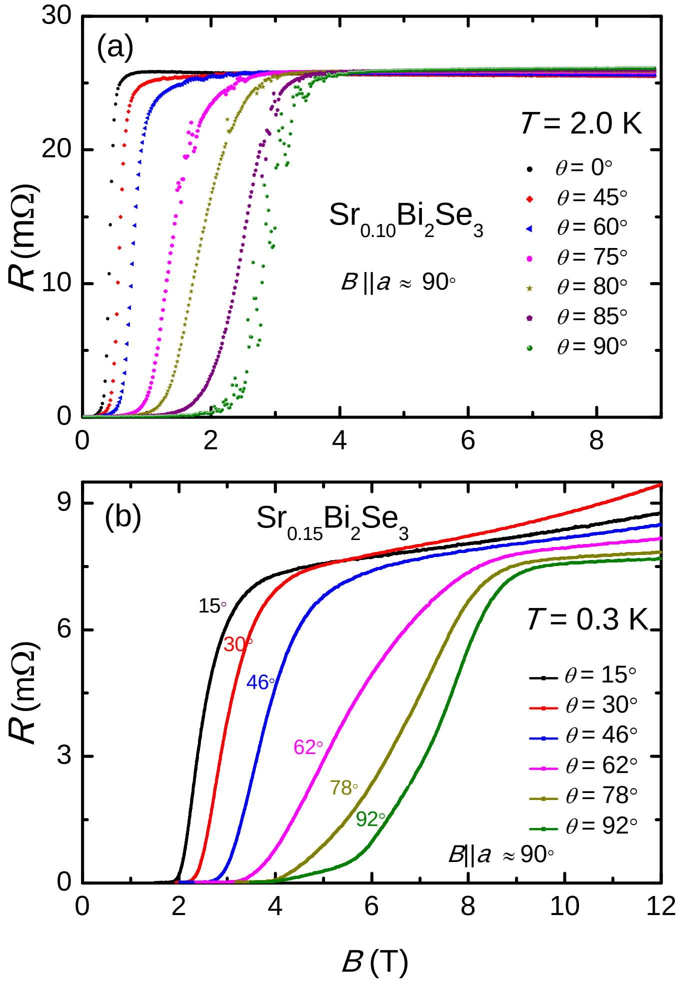

Fig. S7 *Panel (a) and (b): Angular variation of the magnetoresistance of* $Sr_{0.10}Bi_2Se_3$ *at 2.0 K and of* $Sr_{0.15}Bi_2Se_3$ *at 0.3 K, respectively. The angle* $\theta = 0°$ *corresponds to* $B \perp I$. *The angle* $\theta = 90°$ *corresponds to B || I, which is also close to B || a.*

8. Specific heat

The specific heat of a $Sr_{0.15}Bi_2Se_3$ crystal with mass 7.6 mg was measured by the relaxation method using the Heat Capacity Option in the PPMS in the temperature range 2-300 K. In Fig. S8 the data from 2 to 200 K are shown. No sign of a structural transition is found in this temperature range. The data between 200 and 300 K (not shown) show some irregularities that can be attributed to the Apiezon N grease that was used to fix the sample to the specific heat platform (see Heat Capacity Option Manual PPMS 2014, Part Number 1085-150 M5, Quantum Design).

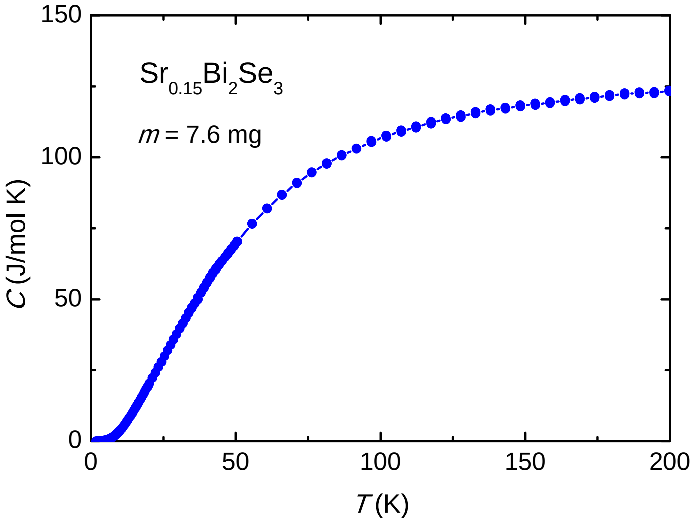

Fig. S8 *Specific heat of* $Sr_{0.15}Bi_2Se_3$ *as a function of temperature.*

9. Current dependence of $R(\theta)$

The angular variation $R(\theta)$ of the $Sr_{0.10}Bi_2Se_3$ crystal was investigated for currents ranging from 0.1 to 2 mA at T = 2 K and B = 1 T. No significant changes are observed for currents $I \leq 1$ mA as shown in Fig. S9. The small increase in resistance for the largest current I = 2 mA is attributed to Joule heating.

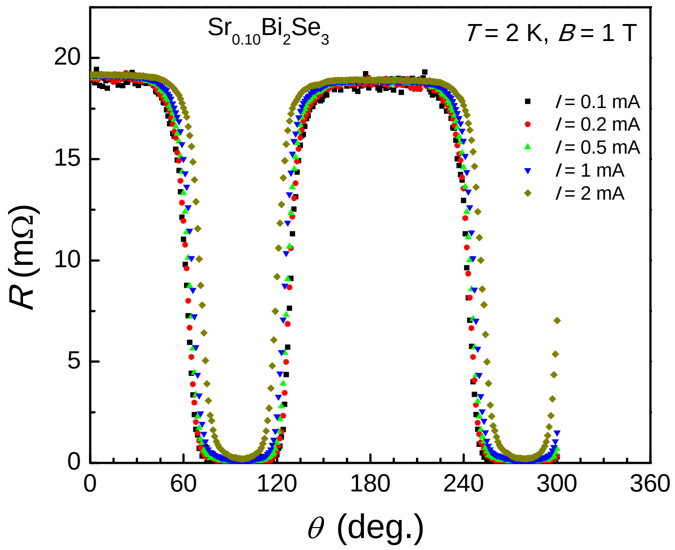

Fig. S9 *Angular variation $R(\theta)$ of $Sr_{0.10}Bi_2Se_3$ measured at T=2 K and B= 1 T for currents ranging from 0.1-2 mA as indicated.*

10. Transport measurements with the current along the *c*-axis

The angular variation $R(\theta)$ in the basal plane was measured with the current along the *c*-axis for $Sr_{0.15}Bi_2Se_3$. In this geometry the *B*-field is always perpendicular to the measuring current. Two circular gold electrodes (diameter 60 μm) were evaporated exactly opposite to each other on the basal-plane surfaces of a thin $Sr_{0.15}Bi_2Se_3$ single crystal (label #2) with dimensions 6×3×0.5 mm^3. Four copper wires (25 μm) that served as current and voltage leads were attached to the electrodes with silver paste, but effectively the resistance was measured in a two-point configuration. Data were taken in the PPMS in the temperature range 2-200 K with a current I = 1 mA, see Fig. S10. Superconductivity results in a drop of 12% of the resistance. In this measurement configuration the transition is relatively broad, The onset temperature for superconductivity for this crystal is 2.75 K. The angular variation for B = 0.5 T in the basal plane is shown in Fig. S11 in the T-range 2.0-3.5 K. The two-fold anisotropy is the same as in the measurements with the current in the basal plane for x = 0.10 (Fig. 1 in the manuscript). For $B \perp I \parallel c$ the lowest resistance values and the largest values for B_{c2} are found for the *B*-field along the long direction of the sample. After the measurements gold electrodes were evaporated at a different place opposite to each other on the basal plane surfaces and the experiment was repeated with essentially the same results and conclusions.

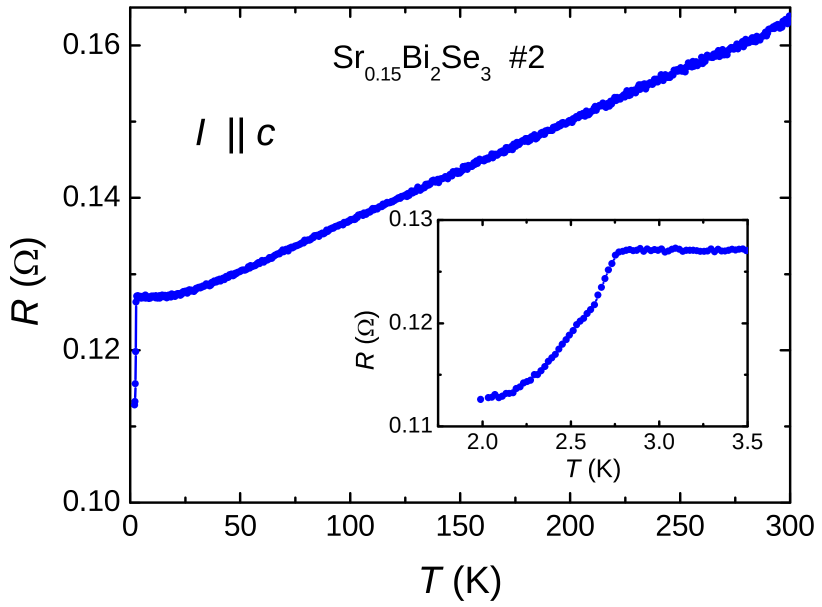

Fig. S10 Resistance of $Sr_{0.15}Bi_2Se_3$ for I || c-axis, measured in a two-point configuration. Superconductivity results in a drop of 12% of the resistance. The residual resistance of 0.112 Ω at 2.0 K is due to the silver paste and the copper wires.

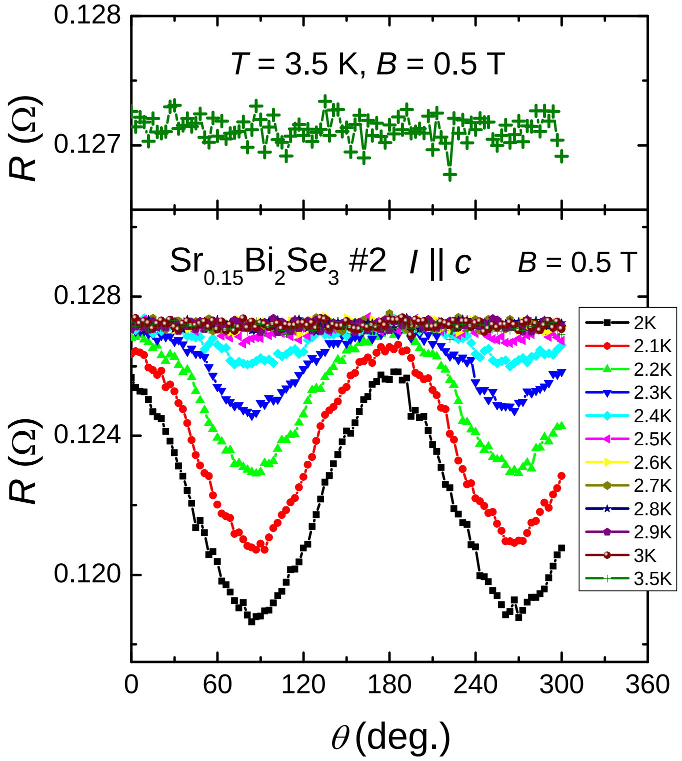

Fig. S11 Lower panel: Angular variation R(θ) in the basal plane for I || c-axis for B = 0.5 T at temperatures as indicated. The lowest curve (black symbols) is at 2.0 K. The anisotropy is two-fold. The lowest resistance values and thus the largest values for B_{c2} *are found for the B-field along the long direction of the sample. Upper panel: Angular variation R(θ) in the normal state for B = 0.5 T. No two-fold oscillation is present within the experimental accuracy.*

In the top panel of Fig. S11 we show the angular variation $R(\theta)$ in the basal plane for B = 0.5 T in the normal state. We remark the resistance is independent of the angle. If the Sr atoms would show some striped ordering one may expect an oscillation in the normal state magnetoresistance. However within this field range and accuracy this is not observed.
